# Investigating the Discriminant Utility of Task-Based and Self-Based Goals in 3 × 2 Achievement Goal Model for Kindergarteners

**DOI:** 10.3390/children9111765

**Published:** 2022-11-17

**Authors:** Chung-Chin Wu

**Affiliations:** Department of Early Childhood Education, National Pingtung University, Pingtung 900391, Taiwan; minin72704@mail.nptu.edu.tw

**Keywords:** achievement goals, kindergartener, mathematics

## Abstract

A decade ago, achievement goal theorists argued that mastery-based goals in the traditional theoretical framework can be theoretically differentiated into task-based goals and self-based goals; and they proposed the 3 × 2 achievement goal model to understand students’ achievement motivation. This new theoretical model has received increasing attention, and it has been demonstrated by several empirical studies on school-aged student samples through analyzing concurrently derived data. Recently, researcher has preliminary demonstrated the new theoretical model on kindergarten sample. It is unclear whether there was a discriminant utility of these goals for kindergartener sample through analyzing their concurrent and predictive effects on learning outcomes. The main purposes of this study were to investigate discriminant utility of task-based goals and self-based goals through examining their concurrent and predictive effects on mathematics performances. A total of 59 kindergarteners aged 5 years consented to participating in this study. Results showed: (1) The discriminant utility of task-approach goal and self-approach goal was only demonstrated on predictive arithmetic performance. (2) The discriminant utility of task-avoidance goal and self-avoidance goal was demonstrated on both concurrent and predictive counting performances. Implications for advancing achievement goal theory, future research, and practice are discussed at the end of the article.

## 1. Introduction

Achievement goals describe the reasons or purposes behind one’s achievement behaviors [1]. Over the past three decades, the achievement goal theory has predominated in the achievement motivation literature. Initially, only two goals, namely the mastery goal and the performance goal, were emphasized. The mastery goal focuses on developing competence through task mastery, whereas the performance goal focuses on demonstrating competence through outperforming others. The performance goal in this dichotomous model is argued to be further differentiated according to approach and avoidance motivation. The trichotomous model, composed of the mastery goal, the performance-approach goal, and the performance-avoidance goal, was proposed and demonstrated [2]. However, the trichotomous model was soon replaced by the 2 × 2 theoretical framework in which the mastery goal was identically differentiated according to approach and avoidance motivation. It resulted in four goals in the 2 × 2 theoretical framework; they were the mastery-approach goal, the mastery-avoidance goal, the performance-approach goal, and the performance-avoidance goal, respectively.

Different achievement goals have been extensively demonstrated to lead to several profound positive or negative effects on mathematics performance. For example, some researchers found that the mastery/mastery-approach goal and the performance-approach goal may have positive effects on achievement, whereas the avoidance-based goals (i.e., the mastery- and performance-avoidance goals) may lead to inadaptive achievement behaviors (i.e., higher test anxiety or lower achievement) [1,2,3,4,5,6,7,8,9,10,11,12,13]. These findings helped teachers above elementary school level to structure their classroom climates and teaching activities to cultivate adaptive achievement goals (e.g., mastery/mastery-approach goals) and sequentially to promote performance [14,15].

Recently, it was argued that the mastery-based goals (i.e., mastery-approach and mastery-avoidance goal) interweaved two different referents (i.e., task requirement and what someone has achieved in the past) to define competence, which may lead to conceptual ambiguity and confound their respective effects on learning outcomes. In addition, there were also several problems in traditional achievement goal measurement. A new 3 × 2 achievement goal model and corresponding measurement were proposed to resolve these problems. The new 3 × 2 achievement goal model structured by the definition and valance of competence, with competence is defined according to three referents (task requirement, self, and other performance) each valanced by approach and avoidance motivations. Consequently, there are six achievement goal dimensions in this model; they are the task-approach, self-approach, other-approach, task-avoidance, self-avoidance, and other-avoidance goals. Task-approach and task-avoidance goals orient someone to achieve and not achieve the task requirements, respectively. Self-approach and self-avoidance goals motivate someone to outperform and to avoid performing poorly than what he/she has achieved in the past, respectively. Other-approach and other-avoidance goals stimulate someone to outperform and to avoid performing inferior to others, respectively [16]. Researchers conducted empirical studies to test this new 3 × 2 achievement goal model through comparing it with several theoretical models (e.g., dichotomous, trichotomous, and 2 × 2 theoretical framework), and the results provided supportive evidence toward this new model. The construct and criterion-related validities of the 3 × 2 achievement goal measurement were preliminary demonstrated on a university education level [16]. After that, supportive evidence related to the construct and criterion-related validity toward 3 × 2 achievement goal theory was sequentially proposed based on school-aged students across countries and subject domains (e.g., engineering and physical education) [17,18,19,20,21,22,23,24,25,26,27,28,29,30,31,32,33,34,35,36,37]. 

Correlations between achievement goals and several outcome variables in the above empirical studies adapting the 3 × 2 achievement goal model are presented in Table 1. There were only task-based goal and self-based goals incorporated in Table 1 because they were interwoven with each other in the traditional theoretical framework and mastery-based goals and performance-based goals have already been demonstrated as different achievement goals since achievement goal theory was proposed [16,38]. Table 1 shows: (1) the discrimination between task-approach goal and task-avoidance goal is relatively clear because they, respectively, have different relationships with 16 outcome variables. (2) Similarly, the discrimination between self-approach goal and self-avoidance goal is generally identified because they also have different relationships with 16 outcome variables. (3) The discrimination between task-approach goal and self-approach goal has also received considerable support because they have different relationships with the 10 outcome variables. (4) In contrast, the discrimination between task-avoidance goal and self-avoidance goal is relatively clear because they also have different relationships with the 15 outcome variables. Taken together, this suggests that mastery-based goals may be differentiated into task-based goals and self-based goals for school-aged students. However, it seems that it is difficult to reveal the discriminant utilities of these goals on their relationships with cognition-related variables (e.g., exam performance and mathematical modelling competency). In addition, the outcome variables were primarily collected concurrently with achievement goals. Consequently, it is unclear if the discriminant utility of task-based goals and self-based goals could be simultaneously supported through their relationships with delayed and concurrent cognitive outcomes for kindergarteners. Recently, the construct validity of the measurement for testing the 3 × 2 theoretical model was preliminary demonstrated on a kindergartener sample [39]. In order to advance the achievement goal theory and, in turn, to benefit mathematics teaching and learning practice in kindergarten, further evidence for supporting the discriminant utility of task-based goals and self-based goals by examining their effects on mathematics performance for kindergarteners is clearly needed.

For above reasons, the purposes of this study are twofold:

- To investigate the discriminant utility of task-based goals and self-based goals for kindergarteners by examining the concurrent effects of which on mathematics performances;
- To clarify the discriminant utility of task-based goals and self-based goals for kindergarteners by examining delayed effects of 3 × 2 achievement goals on later mathematics performances.

## 2. Methodology

### 2.1. Participants

Cluster sampling was firstly used to select classes. Classroom teachers, kindergarteners and their parents in these classes were then invited to participate in this study. Only those participants for whom all three parties gave their agreements were included in this study. Any participants for whom at least one party disagreed were excluded. A total of 59 (29 males and 30 females) kindergarteners aged 5 years, selected from four kindergarten classes in Taiwan, consented to participate in the study. Participants were informed that if they consent to participate all of their responses would be kept strictly confidential, and kindergarteners and their parents were assured that participation would not influence their right to education and treatment by kindergarten teachers.

### 2.2. Instruments

Two instruments for kindergarteners were used in this study; one is pictorial achievement goal measurement and the other is counting and arithmetic test. The test was implemented twice, respectively, at the beginning of and the end of the semester to clarify the utility of discriminating the task-based goals and self-based goals from the mastery-based goals proposed in the 2 × 2 achievement goal framework. 

#### 2.2.1. Pictorial Achievement Goal Measurement for Kindergarteners

Pictorial achievement goal measurement for kindergarteners is currently developed and demonstrated as an equally effective instrument for measuring kindergarten boys’ and girls’ achievement motivation. There were twenty-one pictorial items in the instrument, and they were developed to investigate the six-factor achievement goals for kindergarteners. Each factor was measured by three short stories/items describing the achievement goals related dialogues and behaviors which were observed in learning area in kindergarten classroom. Sample items for each achievement goal factor, respectively, for boys/girls were as follows: (1) John/Mary concentrated on building a castle in the block area (task-approach goal). (2) John/Mary tells the teacher: “I want to build a castle that is higher than I have made in the past” (self-approach goal). (3) John/Mary competes with Tom/Cathy, and says: “I want to build a castle higher than yours” (other-approach goal). (4) John/Mary ran away from the block area because he/she could not build a castle well (task-avoidance goal). (5) John/Mary tells Tom/Cathy: “I do not want to stack up blocks lower than I have made in the past” (self-avoidance goal). (6) John/Mary competes with Tom/Cathy, and says: “I do not want to stack up blocks lower than yours” (other-avoidance goal). Each story description was read out loud to kindergarteners, then they were asked to choose one from the four options on a scale from 1 (“very much unlike me”) to 4 (“very much like me”) scale, each represented by a cartoon face reflecting the extent to which the descriptions of the protagonist in the story was analogous to them [39]. 

#### 2.2.2. Counting and Arithmetic Test for Kindergarteners

A Chinese version of counting and arithmetic test for kindergarteners was developed by partially referring to the test of early mathematics ability (TEMA-3). TEMA-3 was considered as a reliable instrument (with all internal consistency reliabilities are above 0.92) for measuring several mathematics abilities, including numbering, number-comparison, numerical literacy, number facts, calculation skills, and concept understanding, for children between ages of 3 to 8 [41]. 

A Chinese version of counting and arithmetic test for kindergarteners composed of two subtests, respectively, for measuring counting and arithmetic ability was developed. The counting ability subtest consists of eighteen items, including one item for oral counting from 1 to 30, three items for one-to-one correspondence counting within 30, three items for cardinality within 30, three items for numerical literacy within 30, four items for forward verbal counting 30 numbers from specific number within 30, and four items for backward verbal counting from specific number within 30 to 1. The arithmetic ability subtest consists of twenty-two items, including two items for sum (under 10) of two numbers, four items for difference (under 10) between two numbers, four items for addend (within 30) unknown, four items for subtrahend (within 30) unknown, four items for summand (within 30) unknown (4 items), and four items for minuend (within 30) unknown. Consequently, there are 40 items in the Chinese version of counting and arithmetic test. 

The internal consistency reliabilities for counting and arithmetic ability test are, respectively, 0.85 and 0.83 for the first test, and 0.75 and 0.88 for the last test. The overall internal consistency reliabilities for Chinese version of counting and arithmetic test are 0.87 and 0.89 for the first test and the last test, respectively.

### 2.3. Analysis

Scores for each achievement goal items were averaged to form a single indicator to be predicted variable. Similarly, two criterion variables were formed by averaging the scores of two sub-test of counting and arithmetic test. As a result, there were six predicted variables and two criterion variables in each of the two time points. 

**Figure 1:**
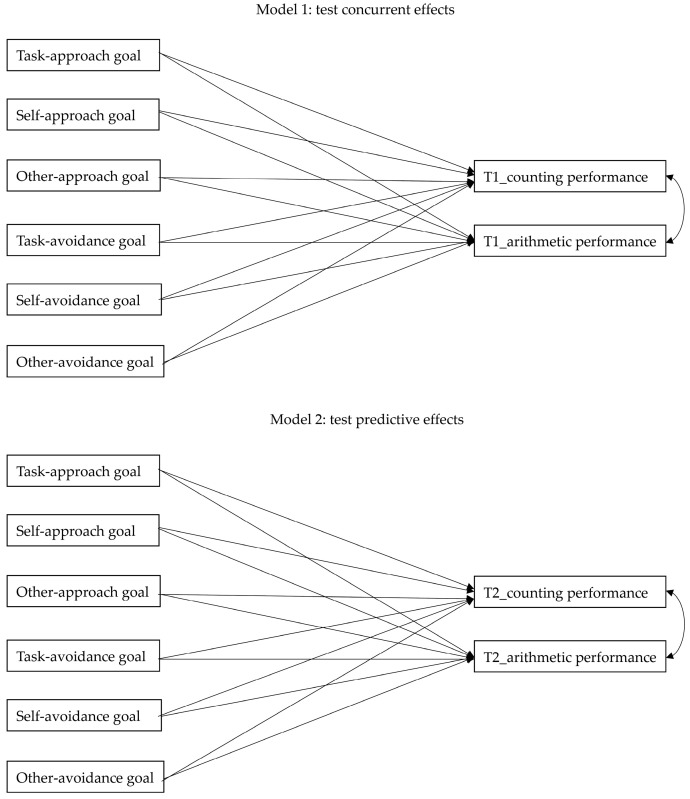
Path models of the effects of achievement goal and mathematics performance. Note. T1 = the first time point of data collection; T2 = the last time point of data collection. For simplicity, intercorrelations among achievement goals are not presented in the Figure.

Two models were proposed, and path analyses were introduced to test concurrent and predictive relationships of task-/self-based goals on mathematics performances. It has to be noted that other-based goals were included in these two models because they may have significant relationships with other goals and influence coefficient estimations. The upper half of the Figure 1 illustrated the predictivities of achievement goals, respectively, on counting and arithmetic performances measured at the beginning of the semester to clarify the concurrent effects of achievement goals. Similarly, the lower half of the Figure 1 illustrated the predictivities of achievement goals, respectively, on counting and arithmetic performances measured at the end of the semester to clarify the predictive effects of achievement goals. All analyses were implemented by using Mplus 7.4, and the maximum likelihood with robust standard errors (MLR) estimator was used to calculate both unstandardized and completely standardized path coefficients. 

The following indices were used to evaluate the model fit: the chi-square statistic (χ^2^), the comparative fit index (CFI), the Tucker–Lewis index (TLI), and the root-mean-square error of approximation (RMSEA). The insignificant chi-square value indicated the model fit the data well. However, the following criteria were also used to evaluate the adequacy of model fit because the chi-square value was sensitive to sample size which made it often reach significant level: CFI and TLI ≥ 0.95, and RMSEA ≤ 0.06 indicated that the model fitted the data very well. 0.90 ≤ CFI < 0.95 and 0.90 ≤ TLI < 0.95, and 0.06 < RMSEA ≤ 0.08 indicated that the model just reached an acceptable level [42,43]. After evaluating the goodness of model fit, both the unstandardized and completely standardized coefficients were reported to investigate the discriminant utility of task-based goals and self-based goals by examining the concurrent and predictive effects of achievement goals on counting and arithmetic performances. 

## 3. Results

### 3.1. Concurrent Effects of Kindergarteners’ Achievement Goals on Mathematics Performances 

Concurrent validity was investigated through analyzing path model 1 in the left side of Figure 1. The results showed that χ^2^(1, *N* = 59) = 0.01, *p* > 0.05, CFI = 1.00, TLI = 3.05, RMSEA = 0.000 (90%CI ranged from 0.000 to 0.087). All these indices met the criteria, which indicated that the path model 1 fit the data very well, and it could be used to explain the predictivities of achievement goals on mathematics performances and to demonstrate the concurrent validity. 

**Figure 2:**
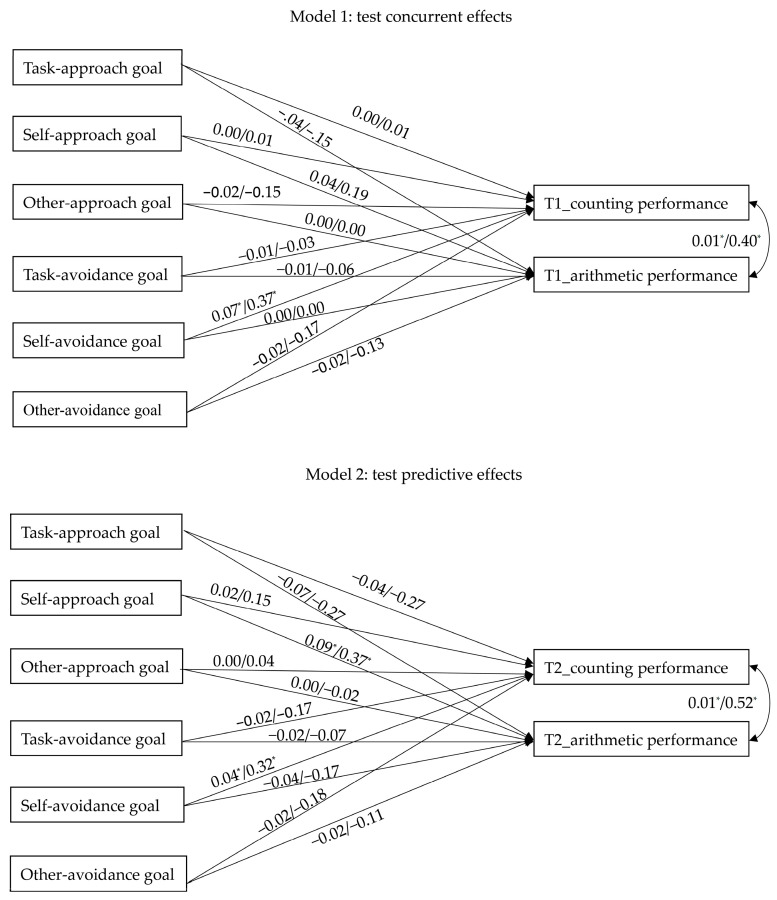
Path coefficients of the concurrent effects and predictive effects models. *Note.* unstandardized coefficients/completely standardized coefficients. For simplicity, intercorrelation coefficients among achievement goals are not presented in the Figure. ^*^ *p* < 0.05.

The path coefficients of two models are simultaneously presented in Table 2 and Figure 2. The results showed that only the self-avoidance goal can positively predict kindergarteners’ counting performance (the unstandardized and completely standardized path coefficients were 0.07 and 0.37, respectively). In contrast, there were no predictivities of other achievement goals on counting performance with the unstandardized and the completely standardized path coefficients ranged from −0.02 to 0.00 and from −0.17 to 0.01 (*p*s > 0.05), respectively. Similarly, all the achievement goals lacked predictivities on arithmetic performance with the unstandardized and the completely standardized path coefficients ranged from −0.04 to 0.04 and from −0.15 to 0.19 (*p*s > 0.05), respectively.

### 3.2. Predictive Effects of Kindergarteners’ Achievement Goals on Mathematics Performances

Predictive validity was examined through analyzing path model 2 in the left side of Figure 1. The results showed that χ^2^(1, *N* = 59) = 0.02, *p* > 0.05, CFI = 1.00, TLI = 1.52, RMSEA = 0.000 (90%CI ranged from 0.000 to 0.168). All these indices were met the criteria, which indicated that the path model 2 fit the data very well, and it could be used to explain the predictivities of achievement goals on mathematics performances and to demonstrate the predictive validity.

The results showed that only the self-avoidance goal can positively predict kindergarteners’ counting performance (the unstandardized and completely standardized path coefficients were 0.04 and 0.32, respectively). In contrast, there were no predictivities of other achievement goals on counting performance with the unstandardized and the completely standardized path coefficients ranged from −0.04 to 0.02 and from −0.27 to 0.15 (*p*s > 0.05), respectively. Similarly, only self-approach goal positively predicts kindergarteners’ arithmetic performance (the unstandardized and completely standardized path coefficients were 0.09 and 0.37, respectively). Other achievement goals lacked predictivities on arithmetic performance with the unstandardized and the completely standardized path coefficients ranged from −0.07 to 0.00 and from −0.27 to −0.02 (*p*s > 0.05), respectively.

## 4. Discussion

The main purposes of this study were to clarify discriminant utility of task-based and self-based goals through examining concurrent and predictive effects of which on mathematics performances. Concurrent and predictive effects of achievement goals on mathematics performances showed supportive evidence toward the discriminant utility between task-based goals and self-based goals. Specifically, there are also no effects of both task-approach and self-approach goals on counting performance measured at T1 and T2 and arithmetic performance measured at T1. Similarly, task-avoidance and self-avoidance goals show no concurrent and predictive effects on arithmetic performance at two time points. These results are identical to studies which investigate the achievement goals on cognitive performance [16,24], and it is also similar to those studies which investigate the achievement goals on other learning outcomes (e.g., satisfaction and exam anxiety) [16,22,24,33]. Seemingly, the results may imply that uncomplicated or/and short-term learning performances have little utility on discriminating task-approach and self-approach goals as well as task-avoidance and self-avoidance goals. In addition, the effects of these achievement goals may be also minimized because the mathematics performances were averaged to form indicators. It may be also suggested that more time was needed for their effects to be presented for a kindergarten sample.

However, for the self-avoidance and task-avoidance goals, the former had a positive effect on counting performance but the latter had no effect; the predictive effects of the task-based and self-based goals on counting performance exhibited the same phenomenon.. These results correspond to studies taking the cognitive variable (e.g., perceived competence) and affective variables (e.g., anxiety) as the outcome [18,19,20,22,24,30,34]. Finally, there is a predictive effect of self-approach goal on arithmetic performance measured at the end of the semester, but the task-approach goal shows no effect. These results are similar to studies which taken energy in class and deep cognitive strategy as outcomes [16,17,18,19,20,21,22,23,24]. It implied that being afraid of performing poorly than what they have achieved in the past may guide kindergarteners to perform better on uncomplicated mathematics skills, and it may have persistent effects. On the contrary, only the active achievement goal that motivate kindergarteners to outperform their past selves had a long-term benefit on skills in complicated mathematics (e.g., arithmetic), and more time may be needed for its effect to appear. 

The discriminant utility of the task-based goal and the self-based goal is partially supported by their distinct effects on mathematics performances for kindergarteners. It seems that their differentiation, based on avoidance motivation, can be seen relatively easily in surface cognitive outcomes (e.g., memorizing or repeating numbers in order). In contrast, their differentiation, based on approach motivation, can be seen relatively easily in deep cognitive outcomes (e.g., understanding or problem solving). It may be also implied that discriminant utility of task-avoidance and self-avoidance goals can be seen relatively easily in their short-term and long-term effects on cognitive outcomes. In contrast, the discriminant utility of task-approach and self-approach goals can be seen relatively easily in their long-term effects on cognitive outcomes.

## 5. Conclusions

For kindergarteners, the discriminant utility of task-avoidance goal and self-avoidance goal can be seen clearly in both short-term and long-term counting performance involving more surface cognitive strategy, but not in both short-term and long-term arithmetic performance involving deeper cognitive strategy. In contrast, the discriminant utility of task-approach goal and self-approach goal may be only revealed in long-term arithmetic performance. This study serves as preliminary evidence which can be used to encourage future studies to further incorporate different cognitive and/or affective outcome variables to re-examine the discriminant utility of both task-based goal and self-based goal on large samples. In practice, the present results suggest that kindergarten teachers can encourage kindergarteners to focus their attention and effort on their own performance to help kindergarteners form self-based goals and in turn to benefit their mathematics performances.

## Figures and Tables

**Table 1:** Empirical status of the correlations between achievement goals and outcome variables.

	TAP	SAP	TAV	SAV
Self-focus anxiety [18]	−	−	+	×
Bodily symptoms anxiety [18]	×	−	×	+
Somatic tension anxiety [18]	−	−	×	+
Perceived control anxiety [18]	+	+	×	−
Worry about exam [18]/[16]	−/×	−/×	+/×	+/×
Exam performance [16]/[20]	×/×	×/−	×/×	×(−)/×
Learning efficacy and absorption in class [16], Emotional recognition [30], and Deep strategy-Relating ideas [24], Task value [20]	+	×	×	×
Energy in class [16]	×	+	×	−
Intrinsic motivation [16]/[34]	+/+	×/+	×/+	×/+
Satisfaction [22]/[31]/[21]	×/+/+	×/+/×	×/+/+	×/+/×
Engagement and positive affect [22], Competence satisfaction [33]	+	+	×	×
Exam anxiety [22]	×	×	+	×
Perceived competence [34], empathy and emotional control-regulation [30] Social attitudes [27], Achievement in social studies [19], Standing longjump [32]	+	+	+	×
Deep strategy-Understanding [24], surface learning strategy [20]	×	+	×	×
Surface strategy-Memorizing [24]	×	×	−	×
Exam performance (Hoi, 2016), Surface strategy-Unreflective studying and executive help- seeking (Hoi, 2016), satisfaction with a win and own performance [33], deep learning strategy [20]	×	×	×	×
Instrumental help-seeking [24]	+	×	×	+
Mathematical modelling competency [23]	+	+	−	−
Problem-solving [40]	−	−	×	-
Entity beliefs [34], 50-m dash [32]	−	−	−	×
Self-efficacy [20]	+	×	×	−
Strategic learning strategy [20]	+	−	×	×
Academic attitudes [27]	+	+	×	+
Engagement [26]	+	+	+	−
Incremental beliefs [34], Physical activity [32], Harmonious passion, obsessive passion, and psychological well-being [35], Mental toughness [17]	+	+	+	+

Note. TAP = task-approach goal. SAP = self-approach goal. TAV = task-avoidance goal. SAV = self-avoidance goal. Symbols in the cells with bold and dark blue highlights indicate discrimination between task-approach goal and self-approach goal in one study. Symbols in the cells with bold and red highlights refer to discrimination between task-avoidance goal and self-avoidance goal in one study. +, −, × represent positive, negative, and no correlation between achievement goals and outcome variables, respectively. × (−) represented results without controlling for response bias.

**Table 2:** Path coefficients of concurrent effect and predictive effect models.

Variables	Task-Approach	Self-Approach	Other-Approach	Task-Avoidance	Self-Avoidance	Other-Avoidance
Model 1						
T1_counting	0.00/0.01	0.00/0.01	−0.02/−0.15	−0.01/−0.03	0.07 */0.37 *	−0.02/−0.17
T1_arithmetic	−0.04/−0.15	0.04/0.19	0.00/0.00	−0.01/−0.06	0.00/0.00	−0.02/−0.13
Model 2						
T2_counting	−0.04/−0.27	0.02/0.15	0.00/0.04	−0.02/−0.17	0.04 */0.32 *	−0.02/−0.18
T2_arithmetic	−0.07/−0.27	0.09 */0.37 *	0.00/−0.02	−0.02/−0.07	−0.04/−0.17	−0.02/−0.11

Note. unstandardized coefficients/completely standardized coefficients. * *p* < 0.05.

## Data Availability

The data presented in this study are available on request from the corresponding author. The data are not publicly available due to research ethics statements which were declared in informed consents.
